# EEG-Based Local–Global Dimensional Emotion Recognition Using Electrode Clusters, EEG Deformer, and Temporal Convolutional Network

**DOI:** 10.3390/bioengineering12111220

**Published:** 2025-11-07

**Authors:** Hyoung-Gook Kim, Jin-Young Kim

**Affiliations:** 1 Department of Electronic Convergence Engineering, Kwangwoon University, 20 Gwangun-ro, Nowon-gu, Seoul 01897, Republic of Korea; 2 Department of Intelligent Electronics and Computer Engineering, Chonnam National University, 77 Yongbong-ro, Buk-gu, Gwangju 61186, Republic of Korea; beyondi@jnu.ac.kr

**Keywords:** emotion recognition, electroencephalography, electrode clusters, EEG deformer, temporal convolution network

## Abstract

Emotions are complex phenomena arising from cooperative interactions among multiple brain regions. Electroencephalography (EEG) provides a non-invasive means to observe such neural activity; however, as it captures only electrode-level signals from the scalp, accurately classifying dimensional emotions requires considering both local electrode activity and the global spatial distribution across the scalp. Motivated by this, we propose a brain-inspired EEG electrode-cluster-based framework for dimensional emotion classification. The model organizes EEG electrodes into nine clusters based on spatial and functional proximity, applying an EEG Deformer to each cluster to learn frequency characteristics, temporal dynamics, and local signal patterns. The features extracted from each cluster are then integrated using a bidirectional cross-attention (BCA) mechanism and a temporal convolutional network (TCN), effectively modeling long-term inter-cluster interactions and global signal dependencies. Finally, a multilayer perceptron (MLP) is used to classify valence and arousal levels. Experiments on three public EEG datasets demonstrate that the proposed model significantly outperforms existing EEG-based dimensional emotion recognition methods. Cluster-based learning, reflecting electrode proximity and signal distribution, effectively captures structural patterns at the electrode-cluster level, while inter-cluster information integration further captures global signal interactions, thereby enhancing the interpretability and physiological validity of EEG-based dimensional emotion analysis. This approach provides a scalable framework for future affective computing and brain–computer interface (BCI) applications.

## 1. Introduction

Emotion is a core psychological phenomenon that significantly influences human cognition, decision-making, and social interaction, and plays a central role in human-centered system design [1]. Consequently, emotion recognition is essential for enhancing responsiveness and adaptability in various applications such as human–computer interaction (HCI), affective computing, digital therapeutics, mental health monitoring, and personalized user interfaces [2]. Traditional approaches have relied on unimodal or multimodal cues, including facial expressions, speech, gestures, and physiological signals, often augmented with artificial intelligence techniques to improve accuracy [3,4]. However, external expressions alone may fail to accurately reflect an individual’s internal emotional state and can be influenced by cultural and situational factors [5].

To overcome these limitations, electroencephalography (EEG)-based emotion recognition has gained attention [6]. EEG provides a noninvasive means to capture emotion-related neural activity with high temporal resolution and real-time analysis. Nonetheless, EEG signals are measured at the electrode level and do not directly reflect the activity of specific cortical regions. They are also characterized by low signal-to-noise ratios, high interindividual variability, and nonlinearity, making accurate emotion classification challenging with simple statistical methods [7]. Conventional electrode-level models achieve only about 70% average accuracy on the SEED dataset [8], while region-based models, which integrate signals from specific scalp areas, reach approximately 78% on the MAHNOB-HCI dataset [9]. Nevertheless, both approaches still face limitations in cross-subject generalization, highlighting the need for a new method that simultaneously integrates local and global EEG patterns.

Emotion arises from interactions among multiple brain regions, forming continuous dimensions that vary with internal states and external stimuli. Accordingly, research distinguishes categorical and dimensional approaches [10]. The categorical approach focuses on classifying discrete emotions such as happiness, sadness, or anger, while the dimensional approach analyzes continuous scales such as valence and arousal to capture the intensity and direction of affective states. This study focuses on dimensional emotion recognition using EEG, which is conceptually distinct from categorical emotion classification.

In this paper, we propose a framework that clusters EEG electrodes based on functional and spatial proximity, extracts local spatiotemporal features from each EEG electrode cluster using an EEG Deformer [11], and integrates these features globally using bidirectional cross-attention (BCA) [12] and a temporal convolutional network (TCN) [13]. This hierarchical design captures long-term temporal dependencies and inter-cluster interactions while systematically modeling electrode-level signal structures rather than replicating actual interregional brain connectivity. The proposed local–global EEG architecture achieves 89% accuracy on unseen subjects, representing an improvement of approximately 19 percentage points (p) over electrode-level models and 11 p over region-based models, demonstrating substantial enhancement in cross-subject generalization.

The main contributions of this study are as follows. First, we propose a hierarchical neural network architecture that models local cluster-level EEG patterns and integrates them for global interaction without mimicking actual brain connectivity, learning statistical dependencies at the electrode-cluster level. Second, in the local cluster feature extraction module, the EEG Deformer is applied to precisely extract spatiotemporal and frequency features. Third, in the global feature integration module, the BCA mechanism captures inter-cluster interactions and long-term temporal dependencies. Fourth, the integrated features at both local and global levels are input to the TCN to learn temporal dynamics and complex relationships among clusters, improving dimensional emotion classification performance.

This approach effectively combines local and global EEG patterns, enhances classification performance, and provides neurophysiologically interpretable representations, offering a practical and valid framework for EEG-based dimensional emotion recognition.

The remainder of this paper is organized as follows. Section 2 reviews related studies. Section 3 describes the proposed methodology. Section 4 presents experimental results. Finally, Section 5 concludes the study and discusses directions for future research.

## 2. Related Works

EEG-based emotion recognition has recently garnered significant attention due to its ability to directly capture neurophysiological activities associated with human emotional states. Early studies primarily relied on handcrafted features, such as band power extracted from specific frequency bands (alpha, beta, gamma), and employed traditional machine learning algorithms, including support vector machines, k-nearest neighbors, and linear discriminant analysis, for emotion classification [14,15]. These approaches typically utilized publicly available EEG emotion datasets, such as DEAP, DREAMER, and SEED. However, handcrafted features are limited in their generalizability and fail to capture the complex spatiotemporal dynamics of EEG signals.

Consequently, deep learning-based approaches have increasingly replaced traditional methods. Convolutional neural networks (CNNs) [16] effectively learn spatial relationships among EEG channels, while recurrent neural network (RNN) architectures, such as long short-term memory (LSTM) networks [17], capture temporal dependencies. Hybrid architectures, such as convolutional RNNs (CRNNs) [18,19], integrate both spatial and temporal features, thereby improving classification performance. The incorporation of attention mechanisms has further enhanced EEG-based emotion recognition [20], allowing models to selectively emphasize emotionally relevant information by weighting specific channels or temporal segments. For example, attention-based convolutional RNNs [21] dynamically adjust the importance of features across channels and time, while three-dimensional convolutional gated self-attention networks [22] integrate complementary information across frequency bands, substantially improving recognition of emotional stress.

Traditional EEG-based models often treat signals as simple multi-channel time series, limiting their ability to reflect the brain’s structural and functional characteristics. Recent studies have attempted to classify EEG electrodes according to major brain regions (frontal, temporal, parietal lobes), learning local features within each region independently and integrating them globally to model the physiological mechanisms underlying emotion generation. However, these approaches are typically conducted at the electrode level, which does not precisely correspond to actual brain regions (source space). As a result, “local learning” and “global integration” may capture statistical dependencies among electrodes rather than true neurophysiological activity. For instance, Zhang et al. [23] proposed an adversarial neural network combined with an attention mechanism to hierarchically learn spatiotemporal EEG features. Wang et al. [24] employed a transformer-based hierarchical model to extend spatial information from electrode-level to brain-region-level representations. Jeong et al. [25] developed a bidirectional gated recurrent unit (BGRU) with self-attention to integrate information between local- and global-level encoders. While these approaches improved recognition accuracy, they remain limited in simultaneously capturing fine-grained local spatiotemporal patterns and global brain-wide interactions.

More recently, methods that segment EEG electrodes based on functional connectivity or anatomical proximity and utilize transformer or graph neural network (GNN) architectures to model inter-regional dependencies have emerged [26,27]. These approaches estimate interaction patterns between brain regions through electrode-level graph representations. Nevertheless, most existing models define graphs solely based on physical adjacency or functional connectivity and often fail to jointly capture fine-grained local spatiotemporal features and global temporal dependencies within a unified framework.

To address these limitations, the present study clusters EEG electrodes according to functional and anatomical proximity, refines local spatiotemporal and frequency features using the EEG Deformer, and integrates them globally through BCA and TCN. This hierarchical framework overcomes the limitations of prior transformer- and graph-based approaches and aims to enhance both the accuracy and neurophysiological validity of EEG-based dimensional emotion recognition.

Furthermore, to overcome the limitations of single-modality EEG, multimodal approaches combining facial expressions, speech, galvanic skin response, and heart rate have been explored [28]. These studies extract emotion-related features across multiple modalities and integrate them using attention-based fusion networks or extended canonical correlation analysis (CCA). For instance, EEGFuseNet [29] and deep generalized CCA with attention mechanisms [30] effectively combine complementary information from EEG and other physiological signals, improving emotion classification performance.

In summary, EEG-based emotion recognition has evolved from statistical approaches to deep learning, transformer, graph, and multimodal methods, yet still faces challenges in jointly modeling local spatiotemporal patterns and global brain dynamics. This study addresses this limitation by integrating EEG Deformer, BCA, and TCN into a hierarchical framework, improving both accuracy and neurophysiological validity.

## 3. Proposed Method

**Figure 1:**
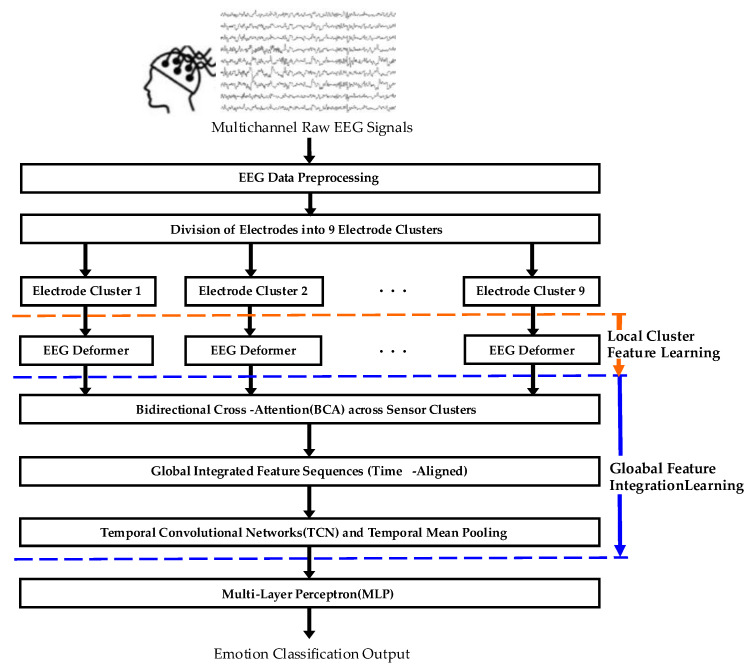
Block diagram of the proposed method.

The proposed EEG-based emotion recognition framework is grounded in the neurophysiological understanding that emotional processes are reflected in spatiotemporal patterns across the scalp rather than in individual electrodes. The model does not assume source-level brain mapping; instead, EEG electrodes are grouped into spatial clusters corresponding to major cortical areas to approximate regional functional organization. This enables the model to learn statistical dependencies among clusters and extract emotion-related patterns at the electrode-space level. The architecture consists of three main components: EEG electrode clustering, localized cluster feature extraction, and global feature integration with dimensional emotion classification, as illustrated in Figure 1.

**Figure 2:**
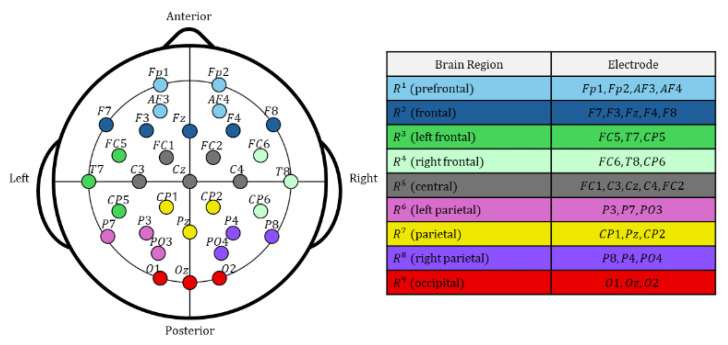
Example of EEG electrode clustering into spatial groups based on approximate correspondence to major cortical regions (32-channel EEG placement using the international 10–20 system). These clusters represent electrode-space groupings rather than direct anatomical brain regions, reflecting statistical dependencies among nearby electrodes.

In the electrode clustering module, scalp electrodes are grouped into nine spatial clusters according to standard EEG electrode placement conventions, capturing statistical dependencies among nearby electrodes and indirectly reflecting functional activities associated with emotional changes [31]. The electrode clusters roughly correspond to prefrontal, frontal, left/right temporal, central, left/right parietal, mid-parietal, and occipital regions (Figure 2) [23]. Multichannel EEG signals within each cluster are input into the localized cluster feature extraction module, the EEG Deformer, which models long-term temporal dependencies and subtle emotional variations through a multi-stage hierarchical structure. Noise and irrelevant signals are filtered out to retain only features relevant to emotion recognition, producing refined cluster-wise feature representations.

The cluster-wise extracted features are then passed to the global feature integration module, where a BCA mechanism enables each cluster to learn statistical interactions with all other clusters. This stage reflects functional dependencies within sensor space and indirectly approximates large-scale brain coordination patterns without assuming direct source-level connectivity. The integrated features are temporally ordered and processed by a TCN to model the temporal evolution of emotional states. The temporally pooled high-dimensional vector is subsequently fed into a multilayer perceptron (MLP) to output the final probability distribution over emotion dimension classes.

Through this hierarchical process, the proposed framework integrates information from local cluster features to global statistical patterns and temporal dynamics, improving the accuracy and stability of EEG-based dimensional emotion recognition.

### 3.1. Localized Feature Extraction at the Electrode-Cluster Level Using EEG Deformer

**Figure 3:**
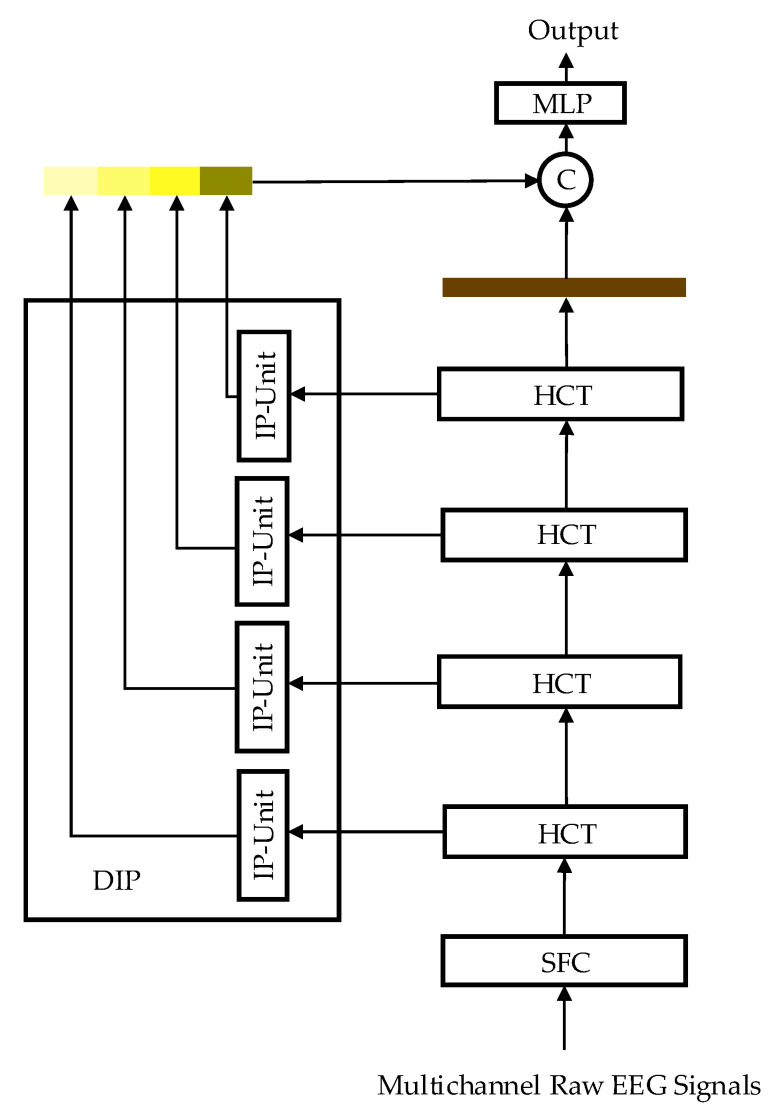
The network structure of EEG-Deformer.

In this study, the EEG Deformer was applied to localized scalp electrode clusters to analyze spatiotemporal signal characteristics and capture subtle EEG variations related to emotional changes. As shown in Figure 3, it comprises three main components: a shallow-feature encoder (SFE), a hierarchical coarse-to-fine transformer (HCT), and a dense information purification module (DIP). The following sections detail each component and their roles in extracting and refining emotion-relevant EEG features at the electrode-space level.

#### 3.1.1. Shallow Feature Encoder (SFE)

EEG signals have a high-dimensional structure defined by time and channel axes and exhibit complex characteristics, such as low SNR, nonlinearity, and spatiotemporally variations. When raw EEG data are fed directly into a self-attention-based transformer architecture, the model may learn irrelevant noise and fail to capture crucial local patterns, resulting in unstable training and degraded performance. To address this, the EEG Deformer first applies a comprehensive preprocessing pipeline before transforming the data through its Shallow-Feature Encoder (SFE).

**Figure 4:**
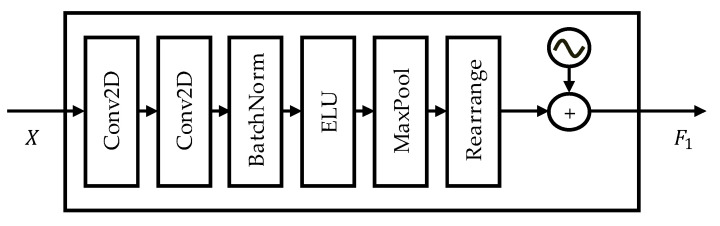
The structure of the shallow feature encoder.

Specifically, the EEG signals are band-pass filtered between 4–47 Hz to remove low- and high-frequency noise, and inter-electrode reference signals are recalibrated to reduce measurement bias. A stacked sparse autoencoder [32] is then employed to detect and remove eye-blink (EOG) and motion artifacts, providing an additional denoising step. The cleaned signals are segmented into 4-s windows and normalized using z-score standardization, resulting in high-quality EEG time-series data. These preprocessed signals are subsequently passed through the SFE, which converts them into low-dimensional, meaningful spatiotemporal representations suitable for input into the hierarchical coarse-to-fine transformer (HCT). As illustrated in Figure 4, the SFE module consists of two sequential two-dimensional convolutional layers (Conv2D), batch normalization (BatchNorm), an exponential linear unit (ELU) [33] non-linear activation, max pooling (MaxPool), rearrangement (Rearrange), and positional encoding [34].

First, the two Conv2D layers capture local dependencies across channels and time steps and simultaneously suppresses irrelevant noise. The subsequent batch normalization step normalizes the distribution of feature maps, thereby enhancing the training stability and convergence speed. The ELU activation function, which is selected by considering the nonlinear characteristics of EEG signals, allows for negative values and mitigates information loss, thereby preserving subtle emotional variations and enabling stable learning. Max pooling reduces the dimensionality of the feature maps while retaining essential information, thereby improving computational efficiency and generalization performance. In the rearrangement step, the features extracted from the CNN are reconstructed into the input format required by the transformer, and positional encoding is added to incorporate the temporal order, enabling the transformer to model sequential dependencies.

Through this process, the SFE extracts meaningful low-dimensional features from complex EEG signals and effectively reduces noise. Thus, the SFE provides a robust foundation for subsequent coarse-to-fine transformers to stably learn long-term dependencies and fine-grained emotional dynamics.

#### 3.1.2. Hierarchical Coarse-to-Fine Transformer (HCT)

**Figure 5:**
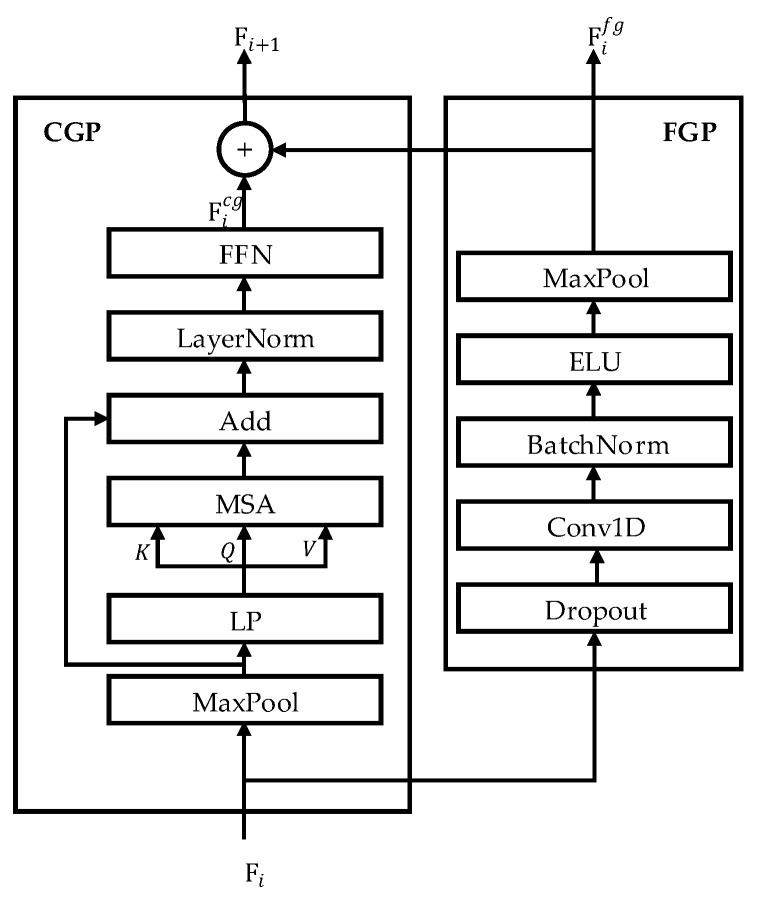
Structure of the hierarchical coarse-to-fine transformer: The left side captures coarse-level temporal information through self-attention, whereas the right side represents the fine-grained temporal learning module.

The EEG Deformer adopts a HCT module to effectively capture short-term fluctuations and long-range dependencies of EEG signals that reflect the dynamic nature of emotions. As shown in Figure 5, this module processes the input EEG features through two parallel temporal pathways (a coarse-grained path and a fine-grained path) to hierarchically learn the spatiotemporal representations related to emotional states.

In the coarse-grained temporal pathway, max pooling is first applied to reduce the temporal resolution, thereby effectively capturing the overall emotional dynamics and long-term trends. Subsequently, a linear prediction operation is applied to maintain temporal continuity in the downsampled sequence. Next, multihead self-attention [21] is used to model long-range dependencies along the temporal dimension. Residual connections and layer normalization (LayerNorm) are incorporated to minimize information loss and ensure stable training. A feedforward network is employed to learn richer and more fine-grained representations.

The embedding generated through this sequence of operations in the coarse-grained pathway (CGP) is denoted $F_{i}^{cg}$ and defined as follows:

(1) $$F_{i}^{cg}=\mathrm{FFN}\left(\mathrm{LayerNorm}\left(\mathrm{MSA}\left(W_{p}\cdot \mathrm{MaxPool}\left(F_{i}\right)+b_{p}\right)+\mathrm{MaxPool}\left(F_{i}\right)\right)\right)$$

where $F_{i}$ denotes the input to the *i*-th HCT block.

By contrast, the fine-grained temporal pathway (FGP) begins by passing the input signals through a dropout layer, which randomly deactivates a subset of neurons to mitigate overfitting. A one-dimensional convolution (Conv1D) is then applied to effectively capture the local temporal dynamics. The resulting features are normalized using batch normalization to improve the training stability, and the ELU activation function is applied to introduce nonlinearity. Subsequently, max pooling is used to decrease the sequence length while retaining the key features. By repeating or hierarchically stacking these operations, the FGP extracts detailed spatiotemporal representations, as expressed by Equation (2).

(2) $$F_{i}^{fg}=\mathrm{MaxPool}\left(\mathrm{ELU}\left(\mathrm{BatchNorm}\left(\mathrm{Convolution}\left(\mathrm{Dropout}\left(F_{i}\right)\right)\right)\right)\right)$$

The outputs of the two paths are then concatenated along the temporal dimension to generate an integrated time-series representation that encompasses both global and local temporal information.

(3) $$F_{i+1}=F_{i}^{concat}=\mathrm{Concat}\left(F_{i}^{cg},{\;F}_{i}^{fg}\right)$$

This fused representation can simultaneously reflect rapid emotional responses and sustained emotional states, thus playing a crucial role in accurately recognizing emotional states from nonstationary EEG signals. The embedding, $F_{i}^{fg}$ is then transmitted to the next stage, that is, the DIP, where meaningful frequency-based emotional information is refined from multilevel features.

Through this process, each layer of the HCT block extracts features at different temporal scales, thereby contributing to the capture of multiscale emotional representations. The lower layers respond sensitively to rapidly changing emotional reactions, whereas the higher layers capture the progression of longer-term emotional states. By hierarchically processing information in this manner, the model effectively reflects the nonstationarity and spatiotemporal variability inherent in EEG signals. In this study, the HCT module was constructed using three layers (*L* = 3), each with the coarse-to-fine parallel structure described above. The gradual increase in the number of layers accounts for the existence of emotional information at various temporal scales: the lower layers focus on rapid emotional responses within relatively shorter time frames, whereas the higher layers capture emotional dynamics across broader temporal ranges. This design strategy extends the typical hierarchical representation-learning structure of transformer-based time-series models to EEG emotion recognition tasks, thereby enhancing the ability of the model to represent emotions across multiple temporal scales.

#### 3.1.3. Dense Information Purification (DIP)

The DIP module is introduced to further refine and integrate the multi-layer fine-grained features $F_{i}^{fg}$ extracted from the HCT blocks. Because of the inherently noisy and spectrally complex property of EEG signals, merely merging features from different layers is insufficient to fully capture the critical information required for emotion recognition. The DIP applies a log-power transform (IP-Unit) *ψ*(·) to the feature maps generated by each HCT layer to overcome this limitation. This transformation accentuates the frequency components that are closely related to emotional states and simultaneously reduces the large variability of the raw signals, thereby improving the stability and reliability of feature representations.

(4) $$P_{i}=\psi \left(F_{i}^{fg}\right)$$

By densely connecting the feature outputs $P_{i}^{b}$ extracted from the *B* HCT blocks, the final DIP representation $F^{DIP}$ is obtained and expressed as follows.

(5) $$F^{DIP}=\mathrm{DenseConcat}\left(P_{i}^{1},\;P_{i}^{2},\ldots ,P_{i}^{B}\right)$$

The DIP module is designed to integrate diverse spatiotemporal and frequency-level features extracted from different layers without loss, enabling multiresolution representations to effectively complement one another. The dense connection structure in DIP also alleviates the vanishing gradient problem that is often observed in deep neural networks, thereby improving the training stability and accelerating convergence. Furthermore, by refining and aggregating rich frequency-domain features across layers, the DIP module generates more expressive representations than single-layer or simply connected architectures. This mechanism plays a crucial role in capturing and interpreting the complex dynamics of EEG signals, ultimately contributing to an enhanced emotion recognition performance.

Finally, the output $F_{i}^{concat}$ from the coarse and fine pathways and the refined representation $F^{DIP}$ obtained through the DIP module are fused and expressed as follows.

(6) $$F_{fused}=\left(F_{i}^{concat},\;F^{DIP}\right)$$

### 3.2. Global EEG Feature Integration via Bidirectional Cross Attention and Temporal Convolutional Network

In this study, the feature vector extracted from each of the nine local EEG electrode clusters is denoted $F_{fused,l}\;\in {\;R}^{d}$, where *l* = 1, 2, …, 9 represents the index of each cluster, and *d* is the dimensionality of each feature vector. These clusters approximate major cortical regions on the scalp, and the extracted features reflect electrode-space statistical patterns rather than direct source-level brain activity.

These vectors are concatenated in the row direction to construct the matrix, $F_{fused}\;\in {\;R}^{9\times d}$:

(7) $$F_{fused}=\left[\left(\begin{array}{c} F_{fused,1}\\ \vdots \\ F_{fused,9} \end{array}\right)\right]$$

It is essential to model not only how each EEG electrode cluster attends to others, but also how it is attended to in return to effectively capture the complex and dynamic statistical interactions among EEG electrode clusters. Therefore, BCA is applied to the matrix, $F_{fused}$, enabling reciprocal interactions among all electrode clusters. First, $F_{fused}$ is projected into Query, Key, and Value matrices using learnable weight matrices $W_{Q},\;W_{K},\;W_{V}\;\in {\;R}^{d\times d_{k}}$ as follows:

(8) $$Q=F_{fused}W_{Q},\;\;\;\;K=F_{fused}W_{K},\;\;\;\;V=F_{fused}W_{V}$$

Next, the similarity score matrix, $S\;\in R^{9\times 9}$, is computed by determining the scaled dot-product of the Query and Key matrices.

(9) $$S=\frac{{QK}^{T}}{\sqrt{d_{k}}}$$

Each element $s_{ij}$ of the score matrix represents the similarity between the query vector of the *i*-th brain region and the key vector of the *j*-th region. The application of the softmax function row-wise yields the attention weight matrix ***α***$\in R^{9\times 9}$:

(10) $$\alpha_{ij=}\frac{exp\left(s_{ij}\right)}{\sum_{k=1}^{9}exp\left(s_{ik}\right)}$$

where $\alpha_{ij}$ indicates the degree of attention that the *i*-th cluster pays to the *j*-th cluster, and $\alpha_{ji}$ reflects the attention in the reverse direction. This bidirectional referencing structure enables each EEG electrode cluster to interact with other clusters, thereby effectively capturing the global statistical interactions among the electrode clusters, without assuming direct source-level brain connectivity.

The attention weight matrix α is multiplied by the value matrix, ***V***, to derive the updated feature matrix, $O\in R^{9\times d_{k}}$:

(11) O=αV

Output ***O*** contains refined representations of each EEG electrode cluster that integrate bidirectional information. These refined features are then arranged temporally to form a global sequence ${\left\{O_{t}\right\}}_{t=1}^{T}$, where T denotes the number of time steps. This sequence serves as the input to a TCN, which uses dilated convolutions to effectively model gradual transitions in emotional states over time.

(12) $$H=\mathrm{TCN}\left({\left\{O_{t}\right\}}_{t=1}^{T}\right)$$

The output of the TCN, ***H***, is then aggregated by average pooling along the temporal dimension to obtain a high-dimensional feature vector, ***h***.

(13) $$h=\frac{1}{T}\sum_{t=1}^{T}H_{t}$$

Finally, ***h*** is fed into an MLP to generate a probability distribution over the dimensional emotion classes.

(14) $$y=\mathrm{softmax}\left(\mathrm{MLP}\left(h\right)\right)$$

Overall, the BCA mechanism effectively captures spatial dependencies among EEG electrode clusters, yielding a high-dimensional representation of their functional relationships. The TCN further integrates and transforms this spatial information over time, enabling the modeling of sequential changes and dynamic emotional transitions. Collectively, these components enable the proposed model to capture complex spatiotemporal patterns in EEG signals at the electrode-cluster level, thereby improving the accuracy and interpretability of emotion recognition.

Algorithm 1 presents a concise overview of our proposed EEG-based local–global emotion recognition pipeline, encompassing preprocessing, electrode clustering, local cluster feature extraction using EEG-Deformer, global feature integration using BCA, and temporal modeling through TCN. Dimensional emotion classification is then performed using an MLP.
**Algorithm 1. EEG-Based Local–Global Dimensional Emotion Recognition**Input: EEG data X ∈ ℝ^(C×T); Cluster groups {C1, C2, …, C9}; Emotion label yOutput: Predicted emotion ŷ**Step 0: EEG Preprocessing**Perform standard EEG preprocessing.Output: Preprocessed EEG data X.**Step 1: EEG Electrode-Clustering**   1.For each EEG sensor s in X: Assign s to cluster Cj according to scalp location.   2.For each cluster Cj: Xj ← EEG_data[sensors ∈ Cj]**Step 2: Local Cluster Feature Extraction (EEG-Deformer)**   3.For each cluster Cj:   4.  a. SFE_out ← SFE(Xj) // Low-dimensional spatiotemporal features   5.  b. HCT_out ← HCT(SFE_out) // Model long-term dependencies and emotional variations   6.  c. Fj ← DIP(HCT_out) // Denoising and feature purification   7.Cluster_features ← {F1, F2, …, F9}**Step 3: Inter-Cluster Interaction Modeling in the Global Feature Integration**   8.G ← BCA(Cluster_features) // Bidirectional Cross-Attention to learn cluster interactions**Step 4: Temporal Dynamics Modeling in the Global Feature Integration**   9.  T ← TCN(G) // Temporal Convolutional Network models emotional dynamics   10.T_avg ← TemporalAveragePooling(T)**Step 5: Dimensional Emotion Recognition**   11.Emotion_pred ← MLP(T_avg)   12.Emotion_distribution ← Softmax(Emotion_pred)   13.ŷ ← argmax(Emotion_distribution)**Step 6: Training Objective**   14.Compute Loss = CrossEntropy(Emotion_distribution, y)   15.Update model parameters via refined multi-stage learning**Step 7: Inference**   16.For a new EEG sample X_new: Repeat Steps 1–4 to predict ŷ

The EEG-Deformer model first extracts initial EEG features using a SFE with a temporal kernel size of Odd (0.1 × fs) and 64 filters. This is followed by a 3-layer HCT with 16 attention heads, a head dimension of 16, an MLP hidden dimension of 16, and a dropout of 0.2 to learn hierarchical temporal patterns. The extracted features are pooled by the DIP module and then processed by a 4-layer BCA mechanism with 16 attention heads, a hidden dimension of 16, a temporal convolution kernel size of 13, and dilation factors of {1,2,4,8}, followed by a FFN with a hidden dimension of 128, GELU activation, layer normalization, and dropout 0.2 to model temporal interactions. The resulting features are further processed by a 3-block TCN with kernel sizes [3,5,7], dilation factors [1,2,4], 64 filters per convolution layer, and dropout 0.2 to learn long-range temporal patterns. After temporal average pooling, an MLP outputs the probability distribution over the class labels. The model is trained using the Adam optimizer (learning rate 0.001, weight decay 1 × 10^−5^, batch size 32, 100 epochs).

## 4. Experiment and Results

The performance of the proposed method was evaluated using three publicly available EEG datasets: DEAP, MAHNOB-HCI, and SEED. These datasets contain EEG signals elicited by audiovisual emotional stimuli, making them suitable for evaluating dimensional emotion recognition models.

### 4.1. Evaluation Datasets

The EEG datasets employed in this study are summarized as follows.

- DEAP [35]: The DEAP dataset was collected from 32 participants (16 males and 16 females) aged 19–37 for emotion recognition research. In addition to EEG, physiological signals such as heart rate and skin conductance were also recorded. Participants watched 40 music video clips while 32-channel EEG signals were recorded at a sampling rate of 512 Hz. Each trial lasted 63 s, including a 3-s baseline period. After each clip, participants rated arousal, valence, dominance, and liking on a 1–9 scale using the self-assessment manikin. Data from 24 participants (12 males and 12 females) with recordings of high quality were retained for analysis.
- MAHNOB-HCI [36]: The MAHNOB-HCI dataset is a multimodal resource that includes EEG and various physiological and visual modalities, such as heart rate, skin conductance, respiration, skin temperature, eye tracking, facial videos, and audio. EEG data were obtained from 27 participants (11 males and 16 females) using 32 channels at a sampling frequency of 256 Hz. The participants viewed 20 video clips (each lasting between 34 and 117 s) designed to elicit diverse emotional responses. After viewing each clip, each participant rated the arousal, valence, dominance, and predictability using a 9-point SAM scale. Data from 22 participants (11 males and 11 females) were used for the final analysis.
- SEED [37]: The SEED dataset comprises multimodal physiological signals, including EEG and eye-movement data, collected from 15 participants (7 males and 8 females). Each participant watched 15 Chinese film clips of about 4 min designed to induce positive, neutral, or negative emotions. EEG was recorded from 62 channels at 1 kHz and downsampled to 200 Hz for analysis. After each clip, participants rated their emotional state on a three-point scale (−1 = negative, 0 = neutral, 1 = positive). Data from 12 participants (6 males and 6 females) with complete sessions and high-quality recordings were included in the analysis.

### 4.2. Models Used for Performance Comparison

The emotion classification performances of the proposed model were determined, and the results were compared with those of models with diverse architectures.

- CNN: This method comprises two convolutional layers with 3 × 3 filters, two 2 × 2 max-pooling layers, a dropout layer, two fully connected layers, and a softmax output layer.
- LSTM: Instead of a CNN, an LSTM network was employed. The first LSTM layer has 128 hidden units, followed by a second LSTM layer with 64 hidden units. This is followed by a dropout layer, a fully connected layer with 128 hidden units, and a softmax output layer.
- CRNN: A model combining a CNN and LSTM was applied to effectively extract spatiotemporal features from multichannel EEG signals. The model consists of two convolutional layers, pooling layers, an LSTM layer, and a final output layer.
- EEGNet [38]: This model is a lightweight CNN architecture that efficiently learns the spatiotemporal features of EEG signals using depthwise separable convolutions. It comprises convolutional blocks, batch normalization, pooling, dropout, fully connected layers, and a softmax output layer.
- EEG Conformer (EEG CF) [39]: This model extracts temporal and spatial features of EEG signals through one-dimensional convolution, learns global temporal dependencies via self-attention, and classifies EEG signals using a fully connected classifier.
- EEG Transformer (EEG TF) [40]: This model is a purely transformer-based architecture that effectively learns long-term dependencies in time-series EEG signals. It comprises embedding layers, positional encoding, self-attention blocks, fully connected layers, and a softmax output layer.
- EEG Deformer (EEG DF): This model extracts temporal features at multiple resolutions directly from multichannel EEG data without dividing the electrodes into predefined spatial clusters, enabling a detailed representation of complex spatiotemporal patterns.
- C2G DF-BCA-TCN: This model is the core method proposed in this study. The model combines an EEG Deformer, BCA, and TCN to extract spatiotemporal features, which are then fed into an MLP for emotion classification.
- C2G DF-TCN: After applying the EEG Deformer to each electrode cluster, the TCN is used without the BCA.
- C2G DF-LSTM: In the C2G EEG DF-TCN structure, LSTM is applied instead of TCN.
- C2G DF-BGRU: In the C2G structure, BGRU [41] is applied instead of TCN to enhance the global spatiotemporal features.

Conventional methods (CNN, LSTM, BGRU, CRNN, EEGNet, EEG CF, EEG TF, and EEG DF) process the entire EEG signal as a single structure. In contrast, C2G architectures (C2G DF-LSTM, C2G DF-BGRU, C2G DF-TCN, and C2G DF-BCA-TCN) divide the EEG electrodes into nine predefined electrode clusters, roughly corresponding to major scalp regions, and extract features based on the electrodes in each cluster.

The performance of each method was evaluated using accuracy. The accuracy was defined as the proportion of correctly classified samples out of the total samples, as expressed in Equation (15).

(15) $$\mathrm{Accuracy}=\frac{\mathrm{TP}+\mathrm{TN}}{\mathrm{TP}+\mathrm{TN}+\mathrm{FP}+\mathrm{FN}}$$

A true positive (TP) counts the samples that are positive and classified correctly, whereas true negative (TN) counts the samples that are negative and classified correctly. A false positive (FP) counts the negative samples that are misclassified as positive, and a false negative (FN) counts the positive samples that are misclassified as negative. Accuracy is the sum of TP and TN divided by the total number of samples.

### 4.3. Experimental Results

In this study, various dimensional emotion-classification experiments were conducted to evaluate the performance of the proposed model. Prior to conducting the experiments, the emotion labels in the DEAP and MAHNOB-HCI datasets were redefined based on rating scores of 1–9 for valence, arousal, and dominance dimensions (Table 1 and Table 2). Valence and arousal were binarized into high and low levels. By combining these two dimensions, four emotion classes were constructed: high valence–high arousal (HVHA), low valence–high arousal (LVHA), low valence–low arousal (LVLA), and high valence–low arousal (HVLA) [39]. This four-class scheme was designed to enable a more fine-grained recognition and classification of emotional states, contributing to improved precision in the emotion inference of the model. In contrast, the SEED dataset categorized emotions into negative, neutral, and positive classes based on valence and was applied directly for a three-class classification without further preprocessing.

Table 3, Table 4 and Table 5 present the results of subject-independent dimensional emotion-classification experiments conducted on the DEAP, MAHNOB-HCI, and SEED datasets, respectively, using two to four emotion classes. All the experiments were conducted using the leave-one-subject-out (LOSO) cross-validation method [42]. In each iteration of the LOSO method, the data from one subject is used as the test set, and the remaining data were used for the training set. This process ensures that each subject’s data are used at least once for testing, with training and testing always performed on data from different individuals. Consequently, the evaluation effectively measured the ability of the model to learn patterns that were generalized to unseen subjects.

The classification accuracy tended to decrease when emotions were classified on the DEAP and MAHNOB-HCI datasets using the valence, arousal, and dominance dimensions (Table 3, Table 4 and Table 5). A decline in performance was observed as the number of emotion classes increased from two to four, likely owing to the increased complexity and variability of the emotional patterns to be learned.

For the DEAP dataset with two and three emotion classes evaluated using the LOSO scheme, eight models were tested: conventional CNN, LSTM, ConvLSTM, Transformer, EEGNet, EEG Conformer, EEG Deformer, and the proposed C2G DF-BCA-TCN (Table 3). These models differed in spatiotemporal feature processing, attention mechanism inclusion, and complexity of their brain-region partitioning and integration approaches.

The C2G DF-BCA-TCN model, which incorporated electrode-cluster-based segmentation and a hierarchical integration structure, achieved the highest classification accuracy, regardless of the number of emotion classes (Table 3). For most models, the classification accuracy ranked in order of dominance, followed by valence, and arousal. In particular, the arousal dimension yielded the lowest performance, which may be because arousal reflects more complex EEG patterns than dominance and valence. The C2G DF-BCA-TCN model achieved the highest accuracy in both binary and three-class classifications, demonstrating superior performance compared to the other models. Although the EEG DF achieves lower recognition accuracy than C2G DF-BCA-TCN, it consistently outperforms other baseline models. This is because, even without explicitly segmenting brain regions at the source level, it effectively extracts fine-grained spatiotemporal features related to emotional dimensions at the sensor-space level through its HCT and DIP modules. In contrast, the EEG Conformer first refines local spatiotemporal features through convolution operations and then integrates global contextual information using a transformer module, resulting in a better representational capability and emotion classification accuracy than EEGNet. Transformer-based models generally outperformed the CNN, LSTM, and CRNN by learning long-range dependencies through self-attention mechanisms. However, the transformer-based models have a structural limitation in that they do not explicitly encode the sequential order of input signals, which is critical for processing time-sensitive biosignals (such as EEG). Because of this limitation, the EEG Conformer exhibited a lower performance than EEGNet, which is more suitable for capturing spatial characteristics and interchannel interactions in EEG signals.

Overall, the experimental results in Table 3 suggest that an integrated model architecture capable of capturing the complex spatiotemporal characteristics of EEG signals plays a crucial role in improving emotion recognition performance. In particular, models with hierarchical integration structures, such as C2G DF-BCA-TCN and EEG Deformer, demonstrate superior performance compared with conventional deep-learning approaches.

The EEG Deformer, which adopts the entire EEG signals as a single structure, were compared with three variants of the proposed C2G DF-BCA-TCN model: C2G DF-TCN, C2G DF-BGRU, and C2G DF-LSTM (Table 4). These variants retain the local cluster-based Deformer architecture but differ in global integration modules. The proposed C2G DF-BCA-TCN model achieved the highest emotion recognition accuracy, followed by C2G DF-TCN, C2G DF-BGRU, C2G DF-LSTM, and the EEG DF (Table 4). Similar to the results in Table 3, in both the two-class and three-class emotion classification tasks on the MAHNOB-HCI dataset, the classification accuracies for dominance and valence were higher than that for arousal.

First, a model architecture that functionally divides the brain into electrode clusters, extracts spatiotemporal information from each cluster via the EEG Deformer, and integrates this information at a global level captures the complex neurophysiological patterns associated with emotional states more effectively than models that process the entire EEG signal as a single undifferentiated structure. Second, although all the compared models exhibited reasonable performance without the application of the BCA technique, the C2G DF-BCA-TCN model significantly improved the emotion recognition accuracy by incorporating BCA. This suggests that optimizing bidirectional interactions between local electrode clusters facilitates information flow across emotion-related clusters, resulting in more accurate and robust representations of emotional states. Third, the structure of the global integration module significantly influences the dimensional emotion recognition performance. Among the compared methods, the TCN-based global integration module (C2G DF-TCN) outperformed the BGRU-based (C2G DF-BGRU) and LSTM-based (C2G DF-LSTM) approaches. This is likely owing to the advantages of the TCN in terms of parallel computation and its ability to effectively and stably capture long-range dependencies. In contrast, although BGRU and LSTM can model temporal sequences, they are less capable of integrating wide temporal contexts and provide limited control over the receptive field sizes, which likely contributes to their relatively lower performance. Overall, these results support the conclusion that model architectures that approximate the structural organization and functional regionalization of the brain are more effective in improving dimensional emotion recognition performance than single-structure-processing approaches. In particular, both the selection of the global integration module (e.g., TCN, BGRU, and LSTM) and the application of the BCA mechanism during the integration of local and global features play critical roles in determining the overall effectiveness of the model.

Table 5 presents the results of the four-class emotion classification experiments conducted on the DEAP and MAHNOB-HCI datasets and the subject-independent three-class classification experiments conducted on the SEED dataset. Among the three datasets, the highest emotion recognition performance was observed for SEED, followed by DEAP and MAHNOB-HCI and DEAP, indicating a decreasing trend in classification accuracy. These performance differences are likely attributed to variations in the dataset structure and data-collection methods. The SEED dataset offered long-duration EEG recordings with repeated sessions from the same participant and high channel density, enabling models to learn the spatiotemporal patterns associated with emotional responses more reliably. In contrast, the DEAP dataset contained shorter EEG segments collected from a diverse group of participants and used subjective self-reported labels, which might lead to decreased consistency in emotional expression, and consequently, reduced recognition performance. The MAHNOB-HCI dataset, which is primarily designed for multimodal analysis, provided relatively limited EEG data in terms of quantity and quality. Furthermore, the restricted number of participants and constrained stimulus conditions possibly hindered the generalization of spatiotemporal pattern learning. In terms of model comparison, the proposed C2G DF-BCA-TCN consistently exhibited the best performance, followed by C2G DF-TCN, C2G DF-BGRU, C2G DF-LSTM, and EEG DF, in descending order. This performance advantage is attributed to the use of the BCA in C2G DF-BCA-TCN, which effectively coordinates the interactions between local electrode clusters and captures complex spatiotemporal patterns in emotional activities of the brain. The differences between the C2G DF-TCN, C2G DF-BGRU, and C2G DF-LSTM reflect their respective global integration strategies based on the TCN, BGRU, and LSTM. In particular, the TCN-based C2G DF-TCN outperformed the other models owing to its parallel processing and stable long-term dependency learning, which are advantageous compared with sequential architectures such as BGRU and LSTM. Therefore, this study confirms that emotion recognition performance depends not only on model architecture but also on experimental factors such as data acquisition, stimulus design, EEG signal quality, and labeling. These results highlight the need to develop robust emotion recognition systems through reliable data collection under controlled conditions and the design of high-performance models.

Table 6 shows the subject-dependent dimensional emotion classification performance of the C2G DF-BCA-TCN model, its variants (C2G DF-TCN, C2G DF-BGRU, and C2G DF-LSTM), and the EEG DF on the SEED, DEAP, and MAHNOB-HCI datasets. In subject-dependent experiments, each model is trained and evaluated separately for each subject, allowing it to capture individual physiological characteristics and typically achieve higher classification accuracy. A paired t-test was conducted to compare the proposed method with other neural network models, with *p*-values below 0.05 indicating statistically significant differences. The results demonstrate that the proposed model achieved average accuracies of 97.5%, 94.3%, and 93.5% for SEED, MAHNOB-HCI, and DEAP, respectively, representing significant improvements over the other models. These accuracies are particularly notable compared with the subject-independent results presented in Table 5, where models are trained without the test subject’s data, reflecting their generalization ability and generally lower performance. Overall, the superior subject-dependent performance indicates that the proposed model is highly effective for personalized dimensional emotion recognition.

**Figure 6:**
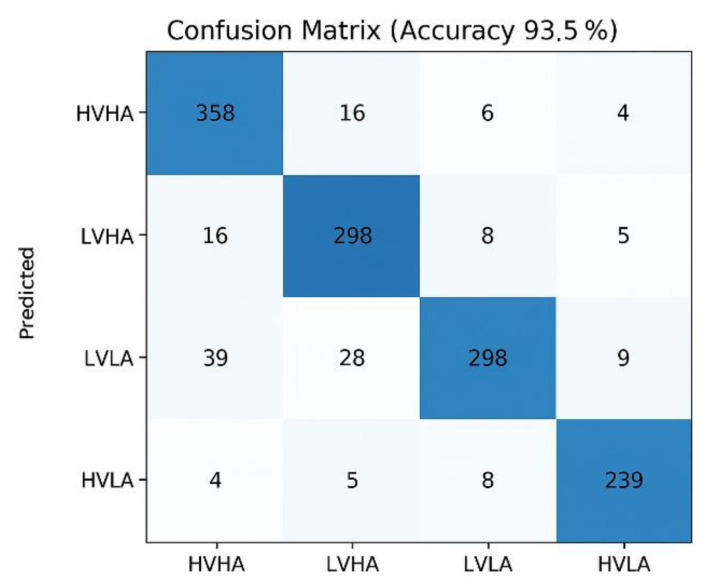
Confusion matrix results of the proposed method applied to the DEAP dataset.

Figure 6 presents the confusion matrix of the proposed method applied to the DEAP dataset, which classifies four emotion states based on valence and arousal (HVHA, LVHA, LVLA, HVLA). Correct classifications are indicated along the main diagonal. The method achieved an overall accuracy of 93.5%, demonstrating robust performance across all emotion states. Specifically, 358 HVHA instances, 298 LVHA instances, 298 LVLA instances, and 239 HVLA instances were correctly classified. Misclassifications were minimal, with most errors occurring between the LVLA and LVHA classes.

The overall integrated architecture demonstrates the effectiveness of the proposed method, but it does not clarify how each component contributes to the final performance. To investigate this, we conducted an ablation study to evaluate the incremental impact of each module—namely, the SFE, HCT, DIP, BCA, and TCN—and this analysis was also applied to the DEAP, MAHNOB-HCI, and SEED dataset. As shown in Table 7, removing DIP from EEG DF led to an average accuracy drop of 6.2% across all three datasets, confirming its critical role in learning stable and reliable representations. Excluding both HCT and DIP resulted in a 13.1% decrease, demonstrating the importance of hierarchical temporal modeling in capturing multi-scale dependencies. When the BCA and TCN modules were removed from the proposed C2G DF-BCA-TCN model, the performance decreased by 5.7%and 2.6%, respectively, indicating that both modules play an essential role in improving the model’s overall effectiveness. Furthermore, when all modules were excluded and only the SFE was applied to the EEG Deformer, the average accuracy dropped by 21.3%, highlighting that each component contributes incrementally to the robustness and precision of the proposed architecture.

### 4.4. Discussion and Study Limitations

This study presents a novel hierarchical framework for EEG-based dimensional emotion recognition, offering meaningful contributions while acknowledging several limitations.

First, the model assigns an individual EEG Deformer to each electrode-space cluster, integrating outputs through the BCA and TCN modules. While this enhances feature integration and classification accuracy, it substantially increases computational complexity and memory requirements, potentially limiting deployment in resource-constrained environments such as real-time mobile BCI systems. Future work should explore model compression techniques, including pruning and knowledge distillation, to mitigate this issue.

Second, the cluster-based approach primarily operates at the electrode-space level, which does not fully correspond to actual source-space brain regions. Consequently, local cluster feature extraction and global feature integration likely capture statistical dependencies among electrodes rather than directly reflecting neurophysiological connectivity. Attention weights learned by the BCA module should therefore be interpreted as electrode-level associations. Future studies should link these patterns to neurophysiological phenomena, including hemispheric dominance, frequency-band relevance, and functional brain networks, and validate the results using source-level analyses or multimodal data (e.g., fMRI, MEG) to enhance interpretability.

Third, EEG electrodes were partitioned into nine fixed clusters. Given inter-individual variability in functional connectivity and emotional responses, static partitioning may limit generalization. Future research should investigate adaptive, subject-specific clustering strategies to improve personalization and capture individual brain dynamics.

Fourth, the EEG datasets used (DEAP, MAHNOB-HCI, SEED) were collected under controlled laboratory conditions. While portable EEG devices offer practical advantages, their reduced electrode count and lower signal quality may affect performance and reproducibility. Developing computationally efficient lightweight models for portable EEG systems is a critical future direction.

Fifth, the emotion labels employed were relatively simple, comprising discrete classes or continuous scales, which may not fully capture the complexity and subjectivity of human affect. Such limitations can lead to overfitting or reduced generalization. This study primarily aimed to validate EEG-based dimensional emotion recognition under subject-independent conditions (LOSO cross-validation), and the experiments serve as an initial evaluation. Future work should incorporate fine-grained continuous labels and ecologically valid datasets to enhance both generalizability and ecological validity.

Finally, while this study did not directly compare with categorical emotion recognition models, its focus was on exploring a dimension-based representation of emotions from EEG signals. Future studies should examine hybrid dimensional–discrete approaches to establish more biophysically and psychologically valid emotion representations.

Importantly, the proposed hierarchical framework not only improves the generalizability of EEG-based emotion recognition but also demonstrates potential applications in digital therapeutics and HCI. For instance, it can support personalized emotion feedback, real-time emotion monitoring, and HCI systems with emotion-adaptive interfaces, among other practical use cases.

## 5. Conclusions and Future Work

In this study, we propose a hierarchical framework that integrates EEG electrode clusters, the EEG Deformer, and TCN. This approach effectively extracts local features from each EEG electrode cluster while also modeling global EEG variations, including inter-cluster correlations. Specifically, the local cluster feature extraction employs the EEG Deformer to capture fine-grained spatiotemporal and frequency features at the electrode-cluster level. Meanwhile, the global spatiotemporal feature integration captures inter-cluster correlations and temporal patterns. Experimental results demonstrate that the proposed model improves dimensional emotion recognition accuracy and interpretability through hierarchical learning that accounts for the structural and functional characteristics of the brain.

However, because the analysis is conducted in electrode space, “local learning” and “global integration” may not fully reflect actual brain source-space activity. Consequently, attention weights learned by the BCA module should be interpreted based on electrode-cluster level correlations. In addition, assigning an individual EEG Deformer to each electrode cluster and integrating outputs via the BCA and TCN modules increases computational complexity and memory requirements. Therefore, future studies should explore lightweight architectures and efficient optimization techniques.

Moreover, the fixed division of EEG electrode clusters may not fully capture inter-individual differences in functional connectivity and emotional responses. To address this limitation, personalized brain-region segmentation and neuroscience-based validation [43,44] should be incorporated to strengthen the physiological validity of learned attention patterns. Development of optimal electrode configurations is also recommended to maintain model performance while ensuring practical applicability. Finally, future research should investigate personalized labeling strategies [45], robust learning methods, and uncertainty-aware modeling techniques [46] to enhance both the accuracy and generalizability of EEG-based emotion recognition systems.

## Figures and Tables

**Table 1:** Emotion classes for two-level emotion classification on DEAP and MAHNOB-HCI datasets.

Rating Values (RVs)	Valence	Arousal	Dominance
1≤RVs≤5	Low	Low	Low
6≤RVs≤9	High	High	High

**Table 2:** Emotion classes for three-level emotion classification on DEAP and MAHNOB-HCI datasets.

Rating Values (RVs)	Valence	Arousal	Dominance
1≤RVs≤3	Negative	Activated	Controlled
4≤RVs≤6	Neutral	Moderate	Moderate
7≤RVs≤9	Positive	Deactivated	Overpowered

**Table 3:** Experimental results of subject-independent dimensional emotion classification using the DEAP dataset.

Methods	Two-Level CL			Three-Level CL		
	VAL	ARO	DOM	VAL	ARO	DOM
CNN	69.7 (11.82)	66.7 (9.50)	70.1 (11.84)	65.3 (10.09)	64.7 (10.43)	65.9 (8.94)
LSTM	75.2 (11.56)	72.3 (10.30)	75.3 (9.11)	71.1 (8.89)	69.7 (11.13)	71.6 (9.46)
CRNN	78.3 (9.26)	77.9 (10.72)	78.5 (11.25)	74.5 (10.56)	73.2 (10.24)	74.8 (10.62)
EEG TF	79.1 (9.57)	78.8 (10.53)	79.6 (9.62)	75.9 (10.26)	75.6 (10.31)	76.3 (10.72)
EEGNet	79.2 (10.53)	77.2 (10.26)	78.3 (9.52)	78.2 (9.57)	75.8 (10.45)	76.6 (10.64)
EEG CF	83.3 (9.86)	80.1 (9.57)	82.8 (10.76)	80.3 (10.62)	78.9 (10.41)	79.3 (10.46)
EEG DF	86.8 (10.45)	85.8 (9.65)	87.6 (9.72)	83.7 (10.64)	82.9 (10.89)	83.5 (10.58)
**C2G DF-BCA-TCN**	**94.7** **(9.24)**	**93.2** **(9.65)**	**94.6** **(10.01)**	**89.5** **(9.32)**	**89.1** **(8.65)**	**89.4** **(9.88)**

Values in parentheses represent the standard deviation. The abbreviations CL, VAL, ARO, and DOM stand for classification, valence, arousal, and dominance, respectively.

**Table 4:** Experimental results of subject-independent dimensional emotion classification using the MAHNOB-HCI dataset.

Methods	Two-Level CL			Three-Level CL		
	VAL	ARO	DOM	VAL	ARO	DOM
EEG DF	87.4 (10.42)	86.5 (9.61)	88.5 (9.85)	81.8 (10.34)	80.8 (10.62)	81.3 (10.73)
C2G DF-LSTM	90.8 (9.78)	89.5 (10.15)	89.7 (9.76)	85.8 (9.68)	85.4 (10.27)	86.5 (9.53)
C2G DF-BGRU	92.1 (9.35)	90.7 (10.24)	91.2 (9.75)	86.9 (10.28)	86.3 (10.38)	87.7 (10.26)
C2G DF-TCN	92.9 (10.45)	91.2 (10.61)	91.9 (9.38)	87.7 (9.65)	87.2 (9.35)	88.4 (10.23)
**C2G DF-BCA-TCN**	**95.4** **(9.27)**	**94.1** **(10.02)**	**95.6** **(9.57)**	**90.1** **(9.63)**	**89.4** **(10.25)**	**90.3** **(9.86)**

Values in parentheses represent the standard deviation. The abbreviations CL, VAL, ARO, and DOM stand for classification, valence, arousal, and dominance, respectively.

**Table 5:** Subject-independent experimental results in four-level classification using DEAP, MANH-OB-HCI dataset, and three-level classification using SEED dataset.

Methods	Four-Level CL (HAHV vs. LAHV vs. HALV vs. LALV)		Three-Level CL (VAL)
	DEAP	MAHNOB-HCI	SEED
EEG DF	77.5 (10.83)	78.1 (9.85)	85.7 (10.68)
C2G DF-LSTM	81.1 (10.56)	81.6 (10.62)	89.4 (11.23)
C2G DF-BGRU	82.3 (9.25)	82.8 (10.83)	90.9 (9.89)
C2G DF-TCN	83.1 (10.12)	83.7 (10.23)	91.7 (9.87)
**C2G DF-BCA-TCN**	**85.3** **(9.25)**	**86.2** **(10.05)**	**94.5** **(9.67)**

Values in parentheses represent the standard deviation. The abbreviations CL, VAL, stand for classification, valence, respectively.

**Table 6:** Within-subject experimental results in four-level classification using DEAP and MAHNOB-HCI datasets and three-level classification using the SEED dataset.

Methods	Four-Level CL (HAHV vs. LAHV vs. HALV vs. LALV)		Three-Level CL (VAL)
	DEAP	MAHNOB-HCI	SEED
EEG DF	85.2 ± 11.5 (0.028)	86.0 ± 10.3 (0.022)	89.2 ± 10.9 (0.018)
C2G DF-LSTM	89.0 ± 10.9 (0.020)	89.6 ± 11.2 (0.016)	92.7 ± 11.7 (0.012)
C2G DF-BGRU	90.2 ± 10.0 (0.016)	91.0 ± 11.0 (0.012)	94.1 ± 10.5 (0.010)
C2G DF-TCN	91.0 ± 10.6 (0.012)	91.7 ± 10.8 (0.009)	94.8 ± 10.2 (0.007)
**C2G DF-BCA-TCN**	**93.5 ± 9.7** **(-)**	**94.3 ± 10.1** **(-)**	**97.5 ± 9.9** **(-)**

Values in parentheses represent the *p*-value. CL, VAL denote classification, valence, respectively.

**Table 7:** Results of the ablation study for within-subject experiments, including four-level classification using DEAP and MAHNOB-HCI datasets, and three-level classification using the SEED dataset.

Methods	Four-Level CL (HAHV vs. LAHV vs. HALV vs. LALV)		Three-Level CL (VAL)
	DEAP	MAHNOB-HCI	SEED
w/o HCT + DIP	71.5 (12.0)	73.1 (11.5)	76.5 (11.0)
w/o DIP	78.7 (11.8)	80.1 (11.0)	82.9 (10.7)
EEG DF	85.2 (11.5)	86.0 (10.3)	89.2 (10.9)
C2G DF-BCA	91.0 (10.6)	91.7 (10.8)	94.8 (10.2)
**C2G DF-BCA-TCN**	**93.5** **(9.7)**	**94.3** **(10.1)**	**97.5** **(9.9)**

Values in parentheses represent the standard deviation. CL, VAL, denote classification, valence, accuracy, and standard deviation, respectively.

## Data Availability

The DEAP dataset can be found at https://www.eecs.qmul.ac.uk/mmv/datasets/deap/ (accessed on 5 July 2023). The MAHNOB-HCI dataset is available online at https://mahnob-db.eu/hci-tagging/ (accessed on 7 July 2023). The SEED dataset is available at https://bcmi.sjtu.edu.cn/home/seed/ (accessed on 8 August 2023).
